# A biodegradable killer microparticle to selectively deplete antigen-specific T cells *in vitro* and *in vivo*

**DOI:** 10.18632/oncotarget.7519

**Published:** 2016-02-17

**Authors:** Wei Wang, Kun Fang, Miao-Chen Li, Di Chang, Khawar Ali Shahzad, Tao Xu, Lei Zhang, Ning Gu, Chuan-Lai Shen

**Affiliations:** ^1^ Department of Microbiology and Immunology, Medical School, Southeast University, Nanjing, PR China; ^2^ School of Biological Sciences and Medical Engineering, Southeast University, Nanjing, PR China; ^3^ Jiangsu Key Laboratory of Molecular and Functional Imaging, Department of Radiology, Zhongda Hospital, Medical School, Southeast University, Nanjing, PR China

**Keywords:** autoimmune disease, allograft rejection, PLGA, p/MHC, alloreactive T cells, Immunology and Microbiology Section, Immune response, Immunity

## Abstract

The specific eradication of pathogenic T cells for the treatment of allograft rejections and autoimmune disorders without impairment of overall immune function is a fundamental goal. Here, cell-sized poly(lactic-co-glycolic acid) microparticles (PLGA MPs) were prepared as a scaffold to co-display the peptide/major histocompatibility complex (pMHC, target antigen) and anti-Fas monoclonal antibody (apoptosis-inducing molecule) for the generation of biodegradable killer MPs. Ovalbumin (OVA) antigen-targeted killer MPs significantly depleted OVA-specific CD8^+^ T cells in an antigen-specific manner, both *in vitro* and in OT-1 mice. After intravenous administration, the killer MPs predominantly accumulated in the liver, lungs, and gut of OT-1 mice with a retention time of up to 48 hours. The killing effects exerted by killer MPs persisted for 4 days after two injections. Moreover, the H-2K^b^ alloantigen-targeted killer MPs were able to eliminate low-frequency alloreactive T cells and prolong alloskin graft survival for 41.5 days in bm1 mice. Our data indicate that PLGA-based killer MPs are capable of specifically depleting pathogenic T cells, which highlights their therapeutic potential for treating allograft rejection and autoimmune disorders.

## INTRODUCTION

Immunosuppressive therapies for allograft rejection and autoimmune diseases are currently being pursued in order to inactivate the entire T cell repertoire, which could lead to overall immune impairment [1-3]. Therefore, new strategies that focus on the selective removal of antigen-specific T cells have become increasingly attractive [4, 5]. One proposed treatment is based on the use of FasL-transfected dendritic cells (DCs) or monocytes to induce peripheral antigen-specific apoptosis of T cells, which are referred to as killer antigen-presenting cells (KAPCs) [6-8]. Despite promising results in the treatment of chronic infections [9, 10], allograft rejection [11], or autoimmune diseases [12], the cell-based KAPCs still suffer from several principal drawbacks: they are time-consuming and cost-intensive when generated on a large scale, and there is batch-to-batch variability in FasL expression. In addition, mature KAPCs may display strong expression of CD86 or 4-1BBL and weak expression of FasL, thereby eliciting vigorous T cell responses towards other antigens and massive neutrophil infiltration [13]. Finally, cellular KAPCs are sensitive to both their *in vivo* and *in vitro* environments due to the activity of cytotoxic T cells, which can lead to KAPC depletion or unwanted changes in cell-cell signaling [14, 15].

To circumvent the limitations associated with the cellular nature of KAPCs, killer artificial antigen-presenting cells (KaAPCs) have been established by covalently coupling the HLA-A_2_-Ig and anti-Fas IgM monoclonal antibody (mAb) onto cell-sized magnetic beads, and were capable of depleting antigen-specific T cells *in vitro* [16]. We previously reported that latex bead-based KaAPCs could selectively deplete 60% alloreactive T cells and prolong alloskin survival for 6 days in a murine model without the loss of overall immune responsiveness [17]. However, despite these promising results, the use of magnetic or latex beads as an acellular scaffold may evoke concerns regarding biosafety and organ toxicity *in vivo.* Therefore, a biodegradable, non-toxic, and biocompatible platform should be further developed.

Polylactic-co-glycolic acid (PLGA) is a biocompatible and biodegradable polymer that has been approved by the United States Food and Drug Administration (FDA) and has been widely used to deliver proteins, small molecule drugs, and other macromolecules in research and clinical settings [18, 19]. In this report, we investigated whether PLGA polyesters could covalently load antigen and monoclonal antibody (mAb) in order to generate killer microparticles (MPs) that could deplete antigen-specific T cells. PLGA MPs with a diameter of 4.0 μm were fabricated on-site using a modified emulsion procedure and co-coupled by H-2K^b^-Ig dimers together with anti-mouse Fas mAbs. Ovalbumin (OVA)_257-264_(SIINFEKL) is a well-known T cell epitope that is presented by H-2K^b^ molecules. Here, it was used as a ‘guided missile’ to target the T cell receptor (TCR) of an OVA_257-264_-specific CD8^+^ T cell clone. Anti-mouse Fas mAb has been shown to induce apoptosis. The killer MPs could efficiently eliminate OVA_257-264_-specific CD8^+^ T cells from transgenic OT-1 mice in an antigen-specific manner *in vitro* and *in vivo*. The retention time and bio-distribution of the killer MPs in mice were also analyzed. Furthermore, killer MPs could deplete H-2K^b^ alloantigen-reactive T cells in H-2K^bm1^ mice, which have a low-frequency of alloreactive T cells, and markedly prolong alloskin graft survival. These data indicate that PLGA MP-based killer MPs are capable of specifically depleting pathogenic T cells, which underscores their therapeutic potential for the treatment of allograft rejections and autoimmune disorders.

## RESULTS

### Characterization of PLGA MPs

**Figure 1:**
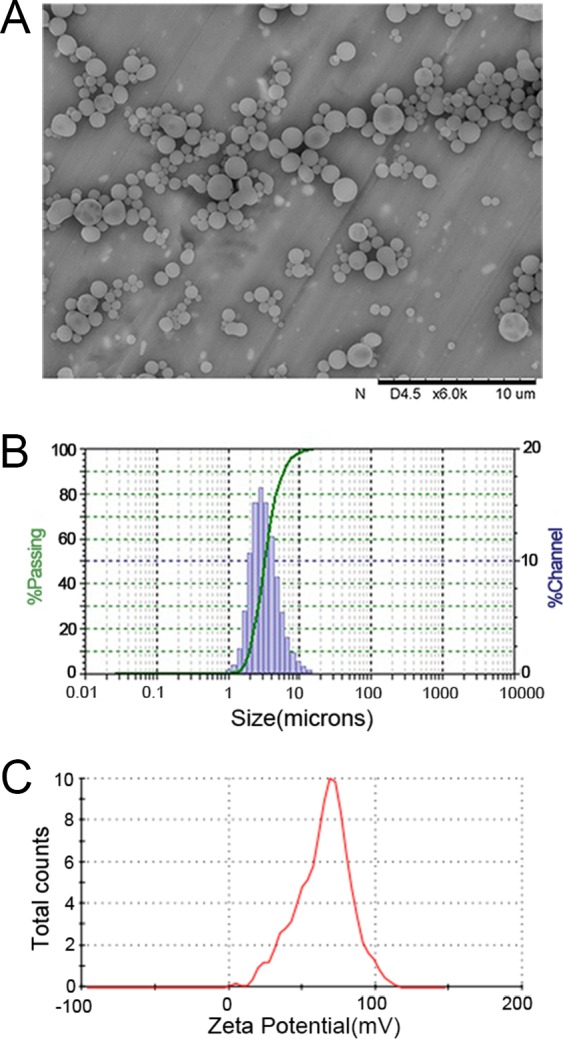
Characterization of PLGA MPs Representative SEM image **A.**, size distribution **B.**, and zeta potential distribution **C.** of PLGA MPs generated on-site.

The PLGA MPs generated here displayed a spherical shape with a smooth surface morphology by scanning electron microscopy (SEM) (Figure 1A). Size analysis revealed diameters that ranged from 1-10 μm, but the diameter of most of the MPs was 4-5 μm (Figure 1B). The mean zeta potential of the MPs was 65.2 ± 6.7 mV as detected using the PALS zeta instrument, suggesting they had a strong capacity to covalently couple proteins (Figure 1C). Indocyanine green (ICG)-encapsulated PLGA MPs exhibited similar characteristics to the PLGA MPs without ICG (data not shown).

### The capacity of PLGA MPs to couple proteins and mAbs

**Figure 2:**
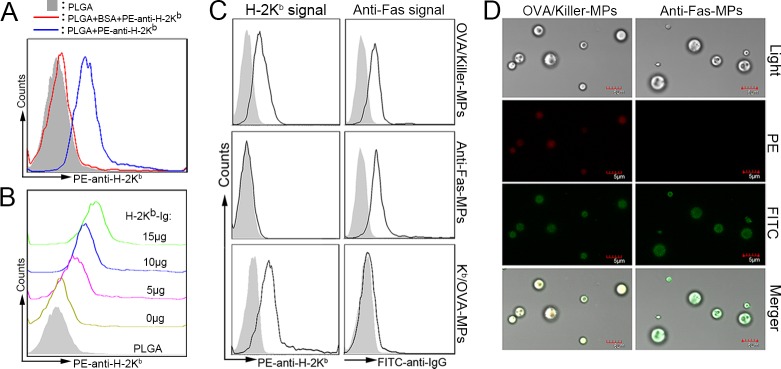
Phenotypic characterization of killer MPs **A.** PE-labeled anti-H-2K^b^ mAbs were coupled to the surface of PLGA MPs and assessed by fluorescence activated cell sorting analysis. **B.** PLGA MPs (1 × 10^8^) were incubated with the indicated amounts of H-2K^b^-Ig dimers overnight, blocked with BSA, and then stained with the PE-anti-H-2K^b^ mAb. **C.**, **D.** Phenotypic characterization of OVA/killer-MPs, anti-Fas-MPs, and K^b^/OVA-MPs. All three types of MPs were stained with PE-labeled anti-H-2K^b^ (clone AF6-88.5) and FITC-labeled anti-hamster IgG (clone G192-1) mAbs, and then analyzed by flow cytometry and confocal laser microscopy. Both H-2K^b^-Ig and anti-Fas were immobilized onto killer MPs, and only anti-Fas and H-2K^b^/OVA were coupled onto anti-Fas-MPs and K^b^/OVA-MPs, respectively (C). Confocal microscopy images confirmed these results (D). The solid histogram in (C) represents the isotype control whereas the solid black line indicates PE-anti-H-2K^b^ or FITC-anti-hamster IgG staining.

To detect whether PLGA MPs were able to load proteins, 5 × 10^6^ MPs were incubated with 1 mL of PBS containing a series of BSA quantities at 4°C overnight with rotation. After centrifugation, retention of the protein in the supernatant was quantified using a Micro BCA Protein Assay. The amount of protein coupled onto the MPs was calculated by subtracting the amount remaining in the supernatant from the original amount in 1 mL of PBS. The maximum amount of protein loaded onto 5 × 10^6^ MPs was approximately 80 μg (data not shown). The MPs were also incubated with a PE-labeled anti-H-2K^b^ mAb to confirm the ability of the MPs to couple mAbs. The MPs displayed a significant fluorescence shift of PE compared to MPs that were blocked with BSA prior to PE-anti-H-2K^b^ mAb coating (Figure 2A). These results demonstrated the strong ability of MPs to couple protein and mAbs.

To determine the appropriate amount of H-2K^b^-Ig used in preparing killer MPs, 1×10^8^ MPs were incubated with the indicated amounts of H-2K^b^-Ig overnight, blocked with BSA, and then stained with a PE-anti-H-2K^b^ mAb. The results showed that the MPs were able to couple H-2K^b^-Ig and displayed a significant fluorescence shift of PE at 10 μg and 15 μg of H-2K^b^-Ig (Figure 2B).

### Phenotypic analysis of killer MPs

Immunofluorescence staining and flow cytometry were used to detect the phenotypes of the killer MPs and control MPs. As shown in Figure 2C, the OVA/killer-MPs displayed both H-2K^b^-Ig and anti-Fas immobilized to the surface of PLGA MPs, whereas the anti-Fas-MPs and K^b^/OVA-MPs only displayed anti-Fas and H-2K^b^-Ig, respectively. Additionally, the fluorescence intensity of anti-Fas on anti-Fas-MPs and H-2K^b^ on K^b^/OVA-MPs were similar to the corresponding signal on OVA/killer-MPs. Confocal images also confirmed the correct phenotype of the OVA/killer-MPs (Figure 2D). Each batch of killer MPs was routinely evaluated in this manner prior to use.

The strong binding of PE-labeled anti-H-2K^b^ mAbs with killer MPs implied that H-2K^b^-Ig dimers coupled onto the PLGA MPs displayed the correct conformation, because the anti-H-2K^b^ mAbs (clone AF6-88.5) recognizes a framework epitope expressed on the β2 m-associated H-2K^b^ heavy chain. The structure recognized outside the peptide-binding site has been shown to be affected by amino acid substitutions in any of the three external domains of the class I heavy chain, and also influenced by the substitution of human for mouse β2 m [20].

### *In vitro* depletion of OVA_257−264_ antigen-specific CD8^+^ T cells by killer MPs

**Figure 3:**
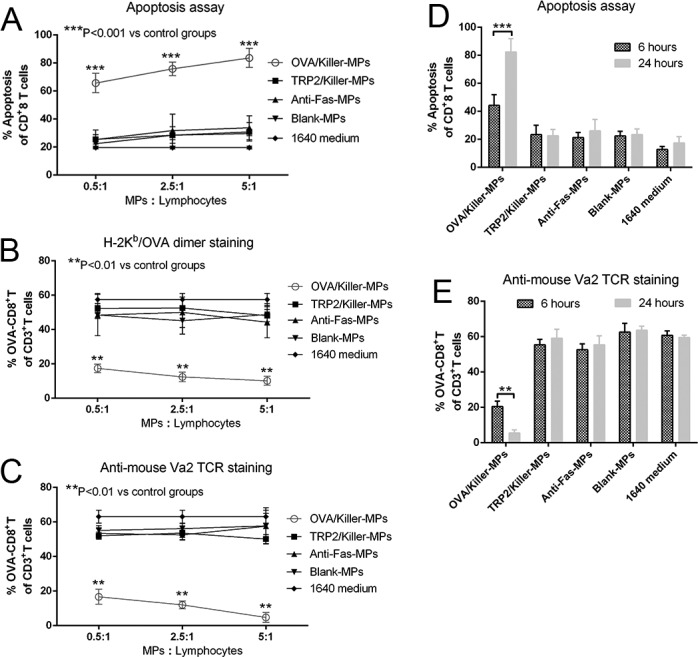
*In vitro* depletion of OVA_257−264_-specific CD8^+^T cells by OVA/killer-MPs **A.**, **B.**, **C.** OVA/killer-MPs exerted a strong apoptotic effect on CD8^+^ T cells and markedly decreased the percentage of OVA_257−264_-specific CD8^+^ T cells in an antigen-specific manner. Lymphocytes from OT-1 mice were seeded into 96-well round-bottom plates and co-cultured with OVA/killer-MPs, TRP2/killer-MPs, anti-Fas-MPs or blank-MPs at various ratios of MPs to lymphocytes. After 24 hours, the co-cultures were stained with annexin V/PI to detect apoptotic CD8^+^ T cells (A), and stained by H-2K^b^/OVA-Ig dimers (B) or anti-mouse Vα2 TCR mAb (C) to detect the frequency of OVA_257−264_-specific CD8^+^ T cells. **D.**, **E.** The killing efficiency of killer MPs increased in an incubation time-dependent manner. Co-cultures were performed for 6 or 24 hours at a 5:1 ratio of MPs to cells. The apoptotic proportion of CD8^+^ T cells (D) and percentage of OVA_257−264_-specific CD8^+^ T cells (E) were detected. ***: *p* < 0.001; **: *p* < 0.01. The data are representative of three independent experiments.

Lymphocytes from OT-1 mice were co-cultured with OVA/killer-MPs, TRP2/killer-MPs, anti-Fas-MPs, or blank-MPs for 24 hours. The killing efficiency was then detected by flow cytometry. Annexin V/propidium iodide (PI) staining revealed a strong apoptotic effect in CD8^+^ T cells induced by OVA/killer-MPs at various ratios of MPs to lymphocytes. In contrast, only a slight apoptotic effect was observed in control co-cultures with TRP2/killer-MPs, anti-Fas-MPs, or blank-MPs, which was comparable to the background death of CD8^+^ T cells cultured alone (Figure 3A). Representative flow cytometric dot plots for each group are shown in Supplementary Figure 1A. The percentage of OVA_257−264_-specific CD8^+^ T cells in the co-cultures with OVA/killer-MPs was remarkably decreased compared to control co-cultures at all ratios of MPs to lymphocytes (Figure 3B and 3C). Notably, TRP2/killer-MPs as an unrelated antigenic epitope control did not lead to an obvious increase in apoptosis and reduction of OVA-specific CD8^+^ T cells, suggesting that the OVA/killer-MPs depleted CD8^+^ T cells in the co-cultures in an antigen-specific manner. Representative flow cytometric dot plots for H-2K^b^/OVA-Ig dimer staining and anti-mouse Vα2 TCR staining in each group are shown in Supplementary Figures 1B and C, respectively. Furthermore, an incubation time-dependent increase in apoptosis and a reduction of OVA_257−264_-specific CD8^+^ T cells was observed in co-cultures with OVA/killer-MPs (Figure 3D and 3E).

**Figure 4:**
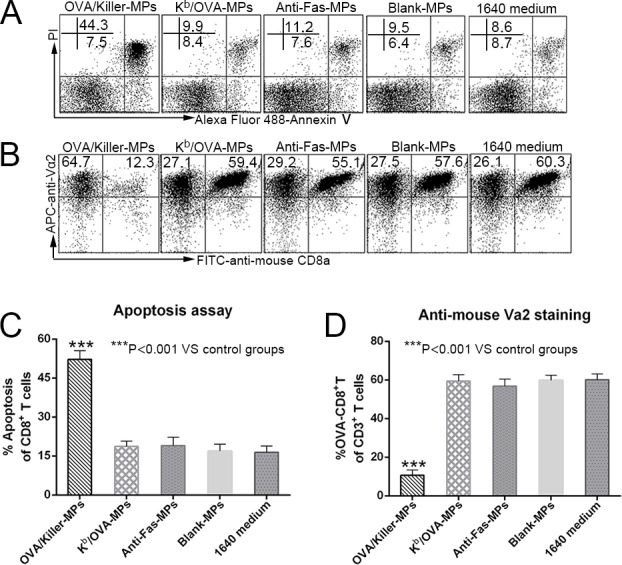
Killer MPs deplete antigen-specific CD8^+^ T cells through Fas/anti-Fas-induced apoptosis Lymphocytes from OT-1 mice were co-cultured with OVA/killer-MPs, K^b^/OVA-MPs, anti-Fas-MPs, or blank-MPs at a 1:1 ratio of MPs to lymphocytes. After 24 hours, the cells were stained with annexin V/PI to detect apoptotic CD8^+^ T cells, and stained with an anti-mouse Vα2 TCR mAb to detect the percentage of OVA_257−264_-specific CD8^+^ T cells. **A.**, **B.** Representative flow cytometric dot plots for apoptosis (A) and anti-mouse Vα2 TCR staining (B). The data are gated on CD8^+^ T cells (A) and CD3^+^ T cells (B), respectively. In contrast to the OVA/killer-MPs, K^b^/OVA-MPs did not result in a significant increase in apoptosis **C.** or a decreased frequency of OVA_257−264_-specific CD8^+^ T cells **D.** in the co-cultures. The data are representative of three independent experiments. ***: *p* < 0.001.

In order to further validate the possible mechanism by which the killer MPs depleted antigen-specific CD8^+^ T cells in co-cultures, K^b^/OVA-MPs were generated as a cognate H-2K^b^-Ig MP control displaying the same amount of K^b^/OVA-Ig but no anti-Fas mAb. Lymphocytes from OT-1 mice were co-cultured with K^b^/OVA-MPs, OVA/killer-MPs, anti-Fas-MPs, or blank-MPs for 24 hours at a 1:1 ratio of lymphocytes to MPs. As shown in Figure 4, K^b^/OVA-MPs, anti-Fas-MPs, and blank-MPs did not result in a significant increase in CD8^+^ T cell apoptosis and decrease in the frequency of OVA-specific CD8^+^ T cells in the co-cultures compared to the 1640 medium group. In contrast, OVA/killer-MPs exerted a strong apoptotic effect resulting in a remarkable reduction in the OVA-specific CD8^+^ T cells. The results revealed that the engagement of a TCR with H-2K^b^/peptide complexes on MPs was not capable of efficiently inducing cell death without Fas/anti-Fas signaling in the co-cultures.

In parallel, the activation processes of OVA-specific CD8^+^ T cells were analyzed by detecting CD44 expression in 6-hour and 24-hour co-cultures. As shown in Supplementary Figure 2, lymphocytes incubated alone in the 1640 medium in the absence of interleukin-2 (IL-2) for 24 hours were only weakly activated, similar to freshly harvested lymphocytes, suggesting that most T cells were in an inactive state before co-culture. IL-2 together with blank-MPs resulted in the activation of more than 50% of the cells, and K^b^/OVA-MPs increased the activation to 25% higher than blank-MPs in the presence of IL-2. These data implied that T cell activation in the co-cultures was the result of synergism between IL-2, MP material, and H-K^b^/OVA target antigens and not only due to the binding of the TCR with the target antigen. Thus, despite the activation in co-cultures, OVA-specific CD8^+^ T cells were not obviously diminished by K^b^/OVA-MPs and other control MPs (Figure 4). The depletion of OVA-specific CD8^+^ T cells by OVA/killer-MPs predominantly contributed to anti-Fas-induced apoptosis, but the possibility of activation-induced cell death (AICD) stimulated by the target antigen could not eliminated.

### *In vivo* depletion of OVA_257−264_ antigen-specific CD8^+^ T cells by killer MPs

**Figure 5:**
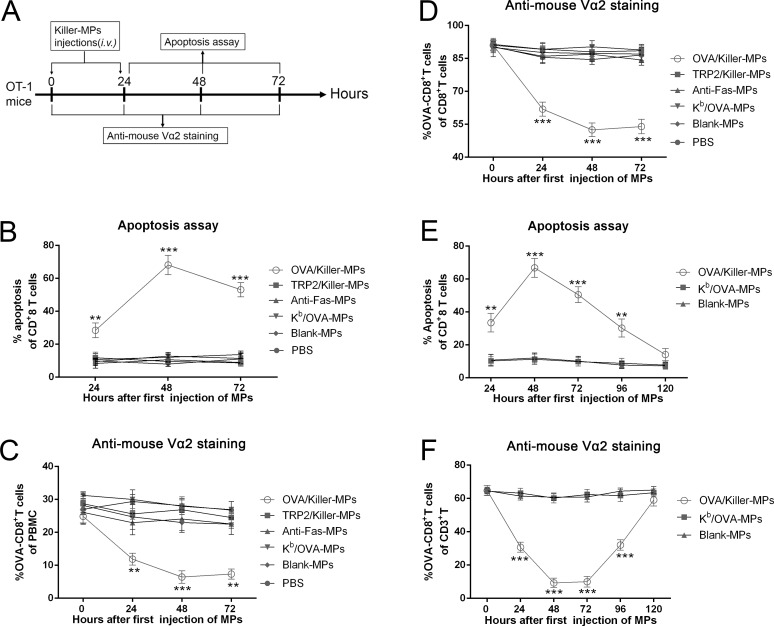
*In vivo* depletion of OVA_257−264_-specific T cells by OVA/killer-MPs **A.** Timeline for *in vivo* depletion. OT-1 mice were injected (*via* the tail vein) with OVA/killer-MPs, TRP2/killer-MPs, anti-Fas-MPs, K^b^/OVA-MPs, blank-MPs, or 0.1 M PBS at 0 and 24 hours. PBMCs were then isolated at various time points and stained with annexin V/PI or an anti-Vα2 TCR mAb. Administration of OVA/killer-MPs elicited a strong apoptotic effect in CD8^+^ T cells **B.** and a marked reduction in OVA_257−264_-specific CD8^+^ T cells among the PBMC **C.** and CD8^+^ T cell populations **D. E.**, **F.** The killing effects of killer MPs persisted for 4 days *in vivo*. OVA/killer-MPs, K^b^/OVA-MPs, or blank-MPs were intravenously administered to OT-1 mice at 0 and 24 hours, and monitored for 120 hours by detecting CD8^+^ T cell apoptosis (E) and the frequency of OVA_257−264_-specific CD8^+^ T cells (F) at various time points. ***: *p* < 0.001; **: *p* < 0.01. Data are representative of two independent experiments (3 mice in each group).

OVA/killer-MPs, TRP2/killer-MPs, anti-Fas-MPs, K^b^/OVA-MPs, blank-MPs, or 0.1 M PBS were administered to transgenic OT-1 mice twice *via* the tail vein at 0 and 24 hours (Figure 5A). Peripheral blood lymphocytes were then isolated at different time points and stained with annexin V/propidium iodide (PI) or an anti-mouse Vα2 TCR mAb. Injection of OVA/killer-MPs resulted in a strong apoptotic effect on CD8^+^ T cells, with the highest percentage (approximately 70%) observed 48 hours after the first injection (Figure 5B). In contrast, the apoptotic effects observed in the TRP2/killer-MPs group and other control groups were within the background level at each time point (Figure 5B). Representative flow cytometric dot plots for each group and time point are shown in Supplementary Figure 3A. The frequency of OVA_257−264_-specific CD8^+^ T cells among both peripheral blood mononuclear cells (PBMCs) (Figure 5C) and CD8^+^ T cells (Figure 5D) decreased substantially after injection of OVA/killer-MPs, but not after injection of TRP2/killer-MPs or other control MPs. Representative flow cytometric dot plots for Figure 5C and 5D are found in Supplementary Figures 3B and 4, respectively. These data demonstrated the ability of killer MPs to selectively deplete antigen-specific T cells *in vivo*.

To investigate how long the depletion effect of OVA/killer-MPs persisted *in vivo*, apoptosis and the frequency of OVA_257−264_-specific CD8^+^ T cells were monitored in the mice for 120 hours. A marked increase in apoptosis (Figure 5E) and reduction of OVA-specific CD8^+^ T cells (Figure 5F) was observed after two injections of OVA/killer-MPs. At the 120-hour time point, both the percentage of apoptotic CD8^+^ T cells and the frequency of OVA-specific CD8^+^ T cells recovered to the levels of the K^b^/OVA-MPs or blank-MPs control groups. The depletion effect appeared to persist for 4 days after the last injection of killer MPs under the present regimen while the K^b^/OVA-MPs were not able to induce significant cell death. Representative flow cytometric dot plots for apoptosis and anti-mouse Vα2 TCR staining are shown in Supplementary Figure 5A and 5B, respectively.

### Near infrared fluorescence imaging

**Figure 6:**
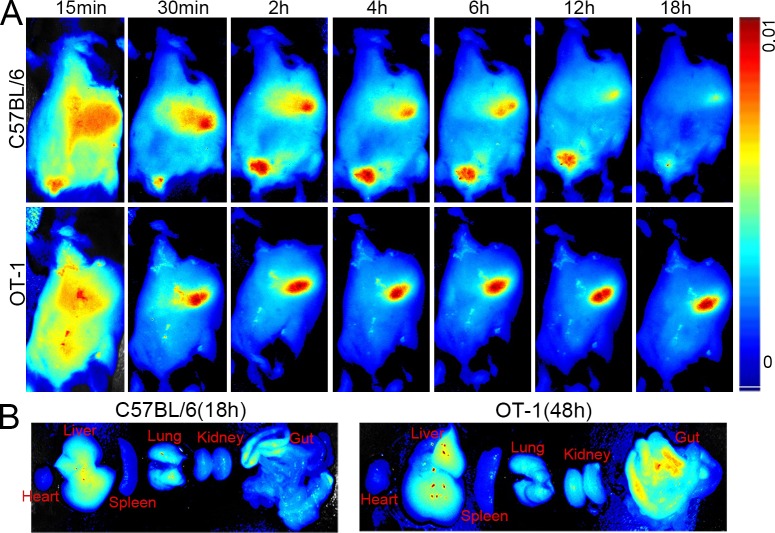
Near infrared fluorescence imaging of killer MPs *in vivo* ICG-encapsulated OVA/killer-MPs were injected (*via* the tail vein) into OT-1 and B6 mice (*n* = 3). Images were then acquired using the Maestro *in vivo* imaging system at different time points. **A.** Representative images of the distributions of ICG-OVA/killer-MPs in tissues of live OT-1 and B6 mice. **B.** The retention of ICG-OVA/killer-MPs in organs excised from OT-1 and B6 mice at indicated time points.

To observe the tissue distribution of killer MPs *in vivo*, OT-1 and C57BL/6 mice were injected *via* the tail vein with ICG-OVA/killer-MPs and then imaged using the Maestro *in vivo* imaging system at various time points. The fluorescent intensity of ICG in C57BL/6 mice was strongest at 6 hours and then decreased with time until 18 hours (Figure 6A, upper panel). However, the fluorescent signal in OT-1 mice was still strong at 18 hours (Figure 6A, lower panel), and was maintained for up to 48 hours (data not shown). Most of the MPs were located in the liver and gut in both the OT-1 and C57BL/6 mice. To further confirm the longer retention time of OVA/killer-MPs in OT-1 mice, endpoint analysis of different organs was performed. As shown in Figure 6B, the fluorescence almost disappeared in excised organs from C57BL/6 mice 18 hours after MP injection, but was still observed in the liver, lungs, and gut of OT-1 mice at the 48-hour time point.

### *In vivo* depletion of H-2K^b^ alloantigen-specific T cells by killer MPs

**Figure 7:**
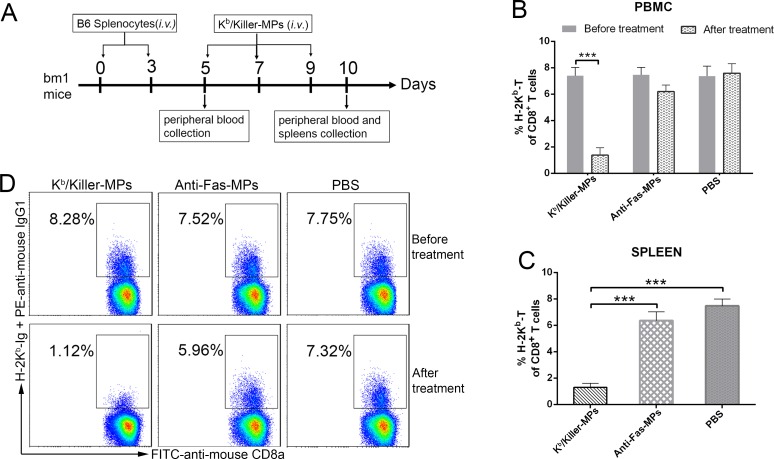
Killer MPs deplete alloantigen-reactive T cells *in vivo.* **A.** Timeline for *in vivo* depletion of H-2K^b^ alloantigen-specific T cells. The bm1 mice were primed twice by splenocytes of C57BL/6 mice on days 0 and 3 (1 × 10^7^ cells/mouse/time point, intravenously), and then injected with K^b^/killer-MPs, anti-Fas-MPs, or PBS on days 5, 7, and 9 (1 × 10^7^ MPs/mouse/time point, intravenously). Peripheral blood was then collected from orbital veins on days 5 (before MP injection) and 10 (after MP injection). The spleens were also collected on day 10. The frequency of H-2K^b^ alloreactive CD8^+^ T cells was detected by H-2K^b^-Ig dimer staining. Administration of K^b^/killer-MPs elicited a marked reduction of H-2K^b^ alloreactive CD8^+^ T cells in the peripheral blood **B.** and spleen **C. D.** Representative dot plots for H-2K^b^-Ig dimer staining in peripheral blood on days 5 and 10. The dot plots are gated on the CD8^+^ T cell population. Data represent the average of three independent experiments (5 mice in each group). ***: *p* < 0.001.

To evaluate the capability of killer MPs to eliminate low-frequency alloantigen-reactive T cells *in vivo*, K^b^/killer-MPs were injected intravenously into bm1 mice (H-2K^bm1^), which were previously primed with splenocytes from B6 (H-2K^b^) mice (Figure 7A). The H-2K^bm1^ allele is a variant that differs from H-2K^b^ by seven nucleotides and results in amino acid substitutions in three codons. The bm1 mice differ from B6 mice only in H-2K^b^ structure. As detected by H-2K^b^-Ig dimer staining and flow cytometry, the percentage of H-2K^b^ alloreactive CD8^+^ T cells in bm1 mice was 7.5 ± 0.51% of the total CD8^+^ T cells in peripheral blood on day 5 (before K^b^/killer-MPs treatment), but markedly decreased to 1.42 ± 0.39% on day 10 (after three injections with K^b^/killer-MPs) (Figure 7B). In parallel, we observed no significant difference in the percentages of H-2K^b^ alloreactive CD8^+^ T cells before and after treatment with anti-Fas-MPs or PBS (Figure 7B). On day 10, the frequency of H-2K^b^ alloreactive CD8^+^ T cells in the spleen was also much lower in the K^b^/killer- MPs group compared to the anti-Fas-MPs and PBS groups (Figure 7C). Representative dot plots for H-2K^b^-Ig dimer staining in the peripheral blood of bm1 mice on days 5 and 10 are shown in Figure 7D. These data demonstrated the ability of killer MPs to eliminate alloantigen-specific T cells *in vivo*.

### Killer MPs treatment prolong alloskin graft survival in a murine model

**Figure 8:**
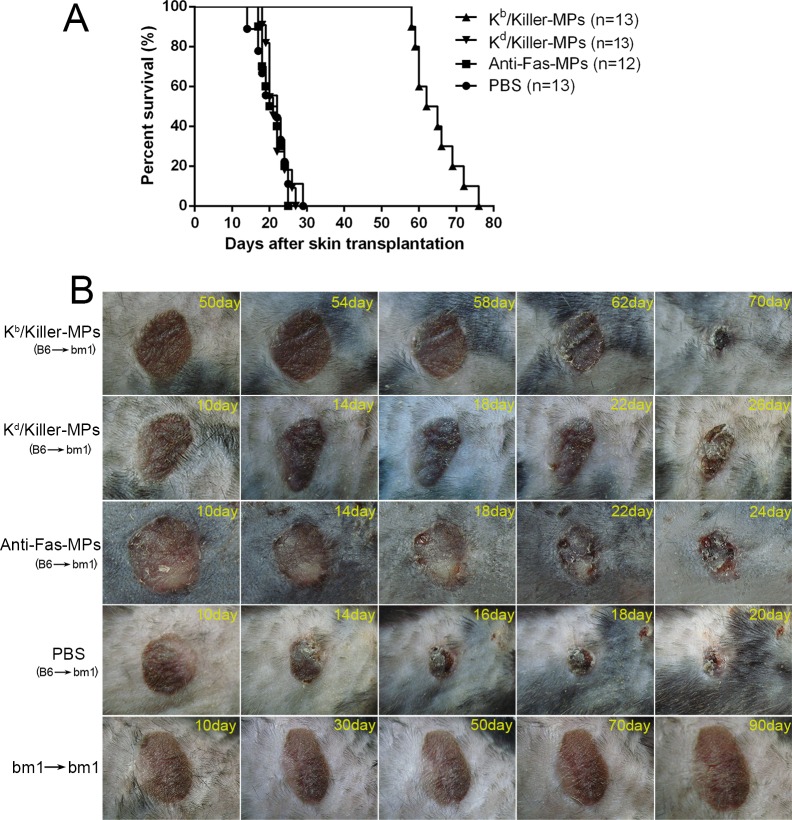
Killer MPs prolong alloskin graft survival in bm1 mice Ear dorsal tissue of C57BL/6 mice was grafted onto the dorsal of bm1 mice. Recipients were then treated (*via* the tail vein) with K^b^/Killer-MPs, K^d^/Killer-MPs, Anti-Fas-MPs or PBS on day 9, 11 and 13 post transplantation (1×10^7^ beads/mouse/time point). The rejection of grafts was monitored daily. Photographs of grafts were taken every two days. **A.** Kaplan-Meier survival plots for skin allograft. The MSTs were 21, 22, 23 and 63.5 days for the PBS, Anti-Fas-MPs, K^d^/Killer-MPs and K^b^/Killer-MPs group, respectively. The differences between the K^b^/killer-MPs group and control groups presented p values less than 0.001, as determined by the log-rank test. **B.** Pictures of representative alloskin grafts on the indicated days. The bm1 autograft was performed to assure a correct procedure of skin transplantation.

To prove the functionality of killer MPs to treat allograft rejections, the K^b^/Killer-MPs were administered intravenously into bm1 mice (H-2K^bm1^) that had previously been grafted with ear skin from C57BL/6 mice (H-2K^b^). The H-2K^bm1^ is a variant allele, which differs from H-2K^b^ by seven nucleotides resulting in amino acid substitutions in three codons. Thus bm1 mice differ from C57BL/6 mice only in H-2K^b^ structure. As shown in Figure 8A, the treatment of K^b^/Killer-MPs prolonged allograft survival for 41.5 days with a median survival time (MST) of 63.5 days. In contrast, the MST of K^d^/killer-MPs, anti-Fas-MPs and PBS group was 23 days, 22 days and 21 days, respectively. The differences between the K^b^/Killer-MPs group and control groups presented p values less than 0.001 as determined by the log-rank test. Notably, K^d^/killer-MPs as a third-party alloantigen control did not lead to an obvious extension of alloskin survival, suggesting that the K^b^/killer-MPs reduced the allograft rejections in the murine model in an antigen-specific manner. Representative pictures for alloskin grafts of each group were shown in Figure 8B. The bm1 autograft was performed to assure a correct procedure of skin transplantation. No rejections were found in the autograft group. These results demonstrated that killer MPs treatment were able to reduce allograft rejection and prolong allograft survival.

## DISCUSSION

PLGA nanoparticles (NPs) and MPs have been widely used to deliver small molecule drugs [21], antigens [22], and siRNA [23], and have been established as safe in humans due to their biocompatible and biodegradable nature [24-27]. Here, we employed PLGA MPs as a scaffold to co-display peptide-MHC molecules (target antigen) and anti-Fas mAb (apoptosis-inducing molecules) in order to generate antigen-specific cytotoxic MPs. OVA antigen-targeted killer MPs could significantly deplete OVA-specific CD8^+^ T cells both *in vitro* and in OT-1 mice in an antigen-specific manner. Moreover, the H-2K^b^ alloantigen-targeted killer MPs were able to eliminate low-frequency alloreactive T cells in bm1 mice and markedly prolong alloskin graft survival. These results demonstrate the therapeutic potential of killer PLGA MPs for allograft rejections and autoimmune disorders.

It is important to note is that this is a preliminary observational study and mechanistic insights are needed. After two injections of killer MPs, not all target T cells in the OT-1 mice were eliminated, and the effects of the depletion persisted for only 4 days. The incomplete effect may have been due to an insufficient dosage, an inappropriate ratio of H-2K^b^ antigens to anti-Fas mAb on killer MPs, or possible limitations in the ability of killer MPs to make contact with T cells in the bloodstream. Thus, the density and ratios of pMHC molecules and anti-Fas mAb immobilized onto MPs should be further titrated to achieve maximal antigen-specific apoptosis of T cells with minimal bystander killing. The route, dosage and frequency of killer MPs administration must be further optimized. Although our data suggest that the deletion of antigen-specific T cells *in vitro* and *in vivo* is due to Fas/FasL-induced apoptosis, we cannot eliminate the possibility that other immunoregulatory mechanisms are involved, such as AICD stimulated by target antigens on killer MPs. One advantage of killer MPs is that one can modulate the signal strength and compose new signal combinations on the surface of PLGA MPs. Currently, we are combining immunoregulatory molecules such as IL-10, TGF-β, and CTLA-4 with anti-Fas to induce more powerful immune inhibition through multiple signal pathways including AICD. Even more, CD47 molecules will be co-coupled onto MPs to prevent the internalization of killer MPs by macrophages *in vivo*.

Near infrared imaging revealed a much longer retention time of OVA/killer-MPs in OT-1 mice than in B6 mice. Whether the high frequency of OVA-specific CD8^+^ T cells resulted in more target killing thereby prolonging the retention of killer MPs in OT-1 mice must be investigated further. The accumulation of MPs in liver, lungs, and gut may correlate with the size and shape of killer MPs. Currently, NPs (< 500 nm) have been extensively applied in targeted drug delivery [28, 29], and are more easily internalized by phagocytes than MPs (∼1-10 μm) [30]. Small MPs (∼1-5 μm) present a reduced risk of engulfment by phagocytic cells and can accumulate in different organs [31]. Additionally, a dramatic effect of shape on attachment and internalization of biomaterials by professional APCs has been demonstrated [32, 33].

Thus far, there has been increasing interest in the application of biomaterials to promote antigen-specific tolerance in autoimmunity and transplantation. After intravenous delivery, PLGA NPs decorated with encephalitogenic peptides were internalized by marginal zone macrophages in the spleen, and induced antigen-specific tolerance in a relapsing experimental autoimmune encephalomyelitis model [34]. Infusions of donor antigen-coupled PLGA NPs (PLGA-dAgs) led to tolerance in approximately 20% of recipient mice in a full MHC-mismatched islet allograft murine model [22]. Moreover, PLGA-NPs containing either protein or peptide and rapamycin [35] as well as gold-NPs with a PEG surface monolayer [36] as vehicles for the delivery of auto antigens to DCs are able to promote the induction of CD4^+^ CD25^+^ Foxp3^+^ regulatory T cells and antigen-specific immunological tolerance. The pMHC-coated iron oxide NPs were capable of depleting activated high-avidity T cells and expanding memory-like autoregulatory T cells, thus preventing type 1 diabetes in non-obese diabetic mice [37]. Unlike these antigen-decorated/coated NPs, which will be mostly internalized by APCs *in vivo*, the cell-sized killer MPs generated here presented both target antigens and immunoregulatory molecules to T cells through direct contact, and could reduce the risk of engulfment by APCs. Thus, they enable depletion of antigen-specific T cells through multiple inhibitory signal pathways, not only through the delivery of antigens to APCs.

In conclusion, our data are the first to demonstrate that PLGA MPs are a viable scaffold for p/MHC molecule and mAb delivery and are capable of inducing antigen-specific T cell apoptosis *in vitro and in vivo*. This novel strategy may represent antigen-specific immunotherapy without overall immune impairment, the major drawback of immunosuppressive agents, for allograft rejections and autoimmune diseases.

## MATERIALS AND METHODS

### Materials

PLGA, a 50:50 poly(lactide-co-glycolide) copolymer (MW 8,000 Da), polyvinyl alcohol (PVA), and ICG (MW774) were purchased from Sigma-Aldrich (St Louis, MO, USA). The H-2K^b^-Ig dimer, H-2K^d^-Ig dimer and purified hamster anti-mouse CD95 mAb (clone Jo2), purified rat anti-mouse CD16/CD32 (clone 2.4G2), PE-labeled mouse anti-mouse H-2K^b^ mAb (clone AF6-88.5), PE-labeled rat anti-mouse IgG1 (clone A85-1), and FITC-labeled mouse anti-Armenian and Syrian hamster IgG mAb (clone G192-1) were purchased from BD Biosciences (Franklin Lakes, NJ, USA). PE-labeled anti-mouse CD4 (clone GK1.5), FITC-labeled anti-mouse CD8a (clone 53-6.7), APC-labeled anti-mouse CD3e (clone 145-2C11), and PE-labeled anti-mouse Vα2 TCR were purchased from eBioscience (San Diego, CA, USA). Polyethylenimine (PEI, 1,800 Da), N-hydroxysuccinimide (NHS, 98%) and N-(3-dimethylaminopropyl)-N- ethylcarbodiimide hydrochloride crystalline (EDC, 99%) were all obtained from Sigma-Aldrich. The Micro BCA Protein Assay kit was purchased from Thermo Fisher Scientific (Waltham, MA, USA). The OVA_257-264_ (SIINFEKL) and TRP2_180-188_ (SVYDFFVWL) peptides were purchased from China Peptides Co. (Shanghai, China), and the purity of each peptide was > 95%.

### Mice

C57BL/6 (H-2K^b^) mice were purchased from the Comparative Medicine Center of Yangzhou University (Yangzhou, Jiangsu, China). Transgenic OT-1 mice were kindly gifted by Dr. Hua Tang (Tai Shan Medical College, Shandong, China). The bm1 (H-2K^bm1^) mice were purchased from the Jackson Laboratory and bred in-house. Mice used in experiments were 8-12 weeks of age. They were maintained in the specific pathogen-free Laboratory Animal Centre of Southeast University (Nanjing, Jiangsu, China). Animal welfare and experimental procedures were performed in accordance with the Guide for the Care and Use of Laboratory Animals (Ministry of Science and Technology of China, 2006) and were approved by the Animal Ethics Committee of Southeast University.

### Fabrication of PLGA MPs and ICG-encapsulated PLGA microparticles (ICG MPs)

MPs were prepared using a double-emulsion solvent evaporation method. PEI was conjugated to the surface of MPs by using modified EDC/NHS chemistry. Briefly, 300 mg PLGA in 2 mL of chloroform with or without ICG was emulsified in 6 mL of 2.5% PVA with a microtip probe sonicator (Microson XL 2000; Misonix Incorporated, Farmingdale, NY, USA) set at level 20 for 30 s. The emulsion was evaporated for 3 hours with magnetic stirring to remove the organic solvent, centrifuged to collect the MPs, and washed twice with water. The MPs were activated using EDC and NHS (Sigma-Aldrich) and were then added dropwise to the PEI (Sigma-Aldrich) solution with magnetic stirring and incubated for another 4 hours at 20°C. Finally, PEI-conjugated MPs were washed twice to remove the free PEI with deionized water.

The resulting MPs were characterized using SEM. The size distribution was analyzed using dynamic light scattering (BI-90 Particle Sizer; Brookhaven Instruments Corporation, Holtsville, NY, USA). The zeta potential of MPs was measured using the ZetaPALS instrument (Brookhaven Instruments Corporation).

### Characterization of the ability of PLGA MPs to couple protein molecules and mAbs

The maximal amount of protein coupled onto MPs was determined using a Micro BCA Protein Assay. Briefly, 5 × 10^6^ MPs were incubated with a series of 1 mL solutions of PBS containing 0 μg, 15 μg, 30 μg, 60 μg, 120 μg, 250 μg, or 500 μg bovine albumin serum (BSA), overnight with rotation at 4°C. After centrifugation at 5000 rpm for 15 min, the supernatant was collected for Micro BCA Protein assays. The amount of protein coupled onto the MPs was then calculated.

To further confirm the ability of MPs to couple proteins such as mAbs, 5 μg of PE-labeled anti-H-2K^b^ mAb was incubated with 2 × 10^6^ MPs overnight with rotation at 4°C. As a control, the MPs were first blocked for 12 hours with 10% BSA in PBS and then incubated with 5 μg of PE-labeled anti-H-2K^b^ mAb as described. After washing twice with PBS, MPs were detected by flow cytometry.

To optimize the amount of H-2K^b^-Ig dimer used in the preparation of killer MPs, 1 × 10^8^ PLGA MPs were incubated with 0 μg, 5 μg, 10 μg, or 15 μg of H-2K^b^-Ig dimers overnight with rotation at 4°C, and then blocked for 12 hours with 10% BSA in PBS. After washing twice with PBS, the MPs were stained with PE-labeled anti-H-2K^b^ mAb for 30 min, and further analyzed by flow cytometry after washing.

### Preparation of antigen-specific killer MPs

OVA_257-264_ (SIINFEKL) is a well-known T cell epitope of OVA and is presented by H-2K^b^ molecules. TRP2_180-188_ (SVYDFFVWL) is an H-2K^b^-restricted, dominant T cell epitope derived from mouse melanoma antigen and was used here as an unrelated antigen control. Killer MPs were prepared by coupling H-2K^b^-Ig (Dimer X, BD) and anti-mouse CD95 (anti-Fas, clone Jo2, BD) mAb onto PLGA MPs. Briefly, 10^8^ MPs were washed twice with sterile 0.1 M PBS and then co-incubated with 10 μg of H-2K^b^-Ig and 10 μg of anti-Fas mAb in sterile 0.1 M PBS at 4°C for 24 hours on a rotator. The killer MPs were then incubated in blocking buffer (0.1 M PBS containing 10% mouse serum) at 4°C for another 24 hours. After washing once with sterile 0.1 M PBS, killer MPs were loaded with either OVA_257−264_ peptide (OVA/killer-MPs) or TRP2_180−188_ peptide (TRP2/killer-MPs) (10 μg/mL) at 4°C for 24 hours and washed twice with sterile PBS before use. In addition, killer MPs containing ICG (ICG/killer MPs) were generated by using the ICG-encapsulated PLGA MPs in the same manner.

In parallel, K^b^/OVA-MPs were generated by coupling 10 μg of H-2K^b^-Ig onto 1 × 10^8^ MPs in the absence of anti-Fas mAb, while anti-Fas-MPs were only coupled with 10 μg of anti-Fas mAb. Blank-MPs were only blocked with BSA and were not incubated with H-2K^b^-Ig or anti-Fas mAb.

In order to analyze the phenotypes of killer MPs, both killer and control MPs were stained with PE-labeled anti-H-2K^b^ mAb and FITC-labeled anti-hamster IgG for 30 min at 4°C in the dark, and acquired on a FACSCalibur flow cytometer (BD Biosciences, San Diego, CA, USA), and analyzed with CellQuest (BD Biosciences) or FlowJo software (BD Biosciences).

### *In vitro* depletion of OVA_257−264_ antigen-specific CD8^+^ T cells by killer MPs

Transgenic OT-1 mice containing transgenic inserts for the murine TCRα-V2 and TCRβ-V5 genes and TCR were designed to recognize OVA residues 257−264 in the context of H-2K^b^, so there was a high frequency of cytotoxic lymphocytes (CTLs) specific for H-2K^b^/OVA_257−264_ complexes in OT-1 mice. Lymphocytes were enriched from the spleens of OT-1 mice and then co-cultured in 96-well round-bottom plates at a density of 2 × 10^5^ cells/well with either control MPs, OVA/killer-MPs, or TRP2/killer-MPs. The ratios of MPs to lymphocytes were 0.5:1, 2.5:1, and 5:1, respectively. Co-cultures were maintained in complete RPMI-1640 medium containing 100 IU/mL IL-2 (BD Biosciences, San Jose, CA, USA) and incubated in a humidified incubator with 5% CO_2_ and at 37°C for 6 or 24 hours. The co-cultures were then collected to analyze the frequency of OVA_257−264_ antigen-specific CD8^+^ T cells and apoptosis by flow cytometry.

### *In vivo* depletion of OVA_257−264_ antigen-specific CD8^+^ T cells by killer MPs

The OVA/killer-MPs, TRP2/killer-MPs, control MPs, or 0.1 M PBS were administered intravenously (*via* the tail vein) to OT-1 mice (1 × 10^7^ MPs in 0.5 mL PBS/mouse). Twenty-four hours later, the administration was repeated as described. To analyze cell survival, 150 μL of peripheral blood was collected from the orbital venous for each mouse at 0, 24, 48, and 72 hours after the first administration. Lymphocytes were then isolated by density gradient centrifugation on Ficoll-Isopaque and the frequency of OVA_257−264_ antigen-specific CD8^+^ T cells and apoptosis detected.

### Near infrared fluorescence imaging

The OVA/killer-MPs encapsulating ICG were injected *via* the tail vein into OT-1 and C57BL/6 mice (1 × 10^8^ MPs/mouse). The mice were then anesthetized by isoflurane inhalation and imaged using the Maestro *in vivo* imaging system (CRi, Woburn, MA, USA) at various time points. Near infrared images were captured at an excitation wavelength of 745 nm and at an emission wavelength of 783 nm with an exposure time of 4 s. At the terminal time point, the heart, liver, spleen, lungs, kidneys, and gut were surgically dissected for *ex vivo* imaging. The imaging parameters were the same as those used for the *in vivo* imaging.

### Detection of the frequency of OVA_257−264_-specific CD8^+^ T cells and apoptosis

Two specific reagents (an anti-mouse Vα2 TCR mAb and H-2K^b^/OVA_257−264_-Ig dimer) were used to detect OVA_257−264_-specific CD8^+^ T cells. Co-cultures or lymphocytes isolated from peripheral blood or splenocytes from OT-1 mice were stained with FITC-labeled anti-mouse CD8a, APC-labeled anti-mouse CD3e and PE-labeled anti-mouse Vα2 TCR for 30 min at 4°C in the dark. For H-2K^b^/OVA_257−264_-Ig dimer staining, co-cultures or lymphocytes were first blocked with anti-mouse CD16/CD32 (clone 2.4G2) for 30 min and then incubated with a mixture of H-2K^b^/peptide-Ig dimers and PE-labeled anti-mouse IgG1 (clone A85-1) for 1 hour at 4°C in the dark. After washing, FITC-labeled anti-mouse CD8a and APC-labeled anti-mouse CD3e were added and the mixture incubated for an additional 30 min. Finally, cells were washed twice with 0.1 M PBS and analyzed using a FACSCalibur flow cytometer (BD Biosciences, San Diego, CA, USA) and analyzed with CellQuest or FlowJo software (BD Biosciences).

Annexin V and PI staining was performed to detect apoptotic cells. Apoptosis assays were performed according to the manufacturer's protocol (Dead Cell Apoptosis Kit, Invitrogen, Carlsbad, CA, USA) and analyzed by flow cytometry as described.

### *In vivo* depletion of H-2K^b^ alloantigen-specific T cells by killer MPs

H-2K^b^ alloantigen-targeted K^b^/killer-MPs were prepared by co-coupling 10 μg of the anti-Fas mAb and 10 μg of peptide-unloaded H-2K^b^-Ig dimers onto 1 × 10^8^ PLGA MPs as described. Splenocytes from C57BL/6 (H-2K^b^) mice were collected and injected into bm1 mice (H-2K^bm1^) *via* the tail vein on days 0 and 3 (1 × 10^7^ cells/mouse/time point). All of the primed bm1 mice were then randomly assigned to one of three groups and injected (*via* the tail vein) with K^b^/killer-MPs, anti-Fas-MPs, or 0.1 M PBS on days 5, 7, and 9 (1 × 10^7^ MPs/mouse/time point).

To detect the frequency of H-2K^b^ alloantigen-reactive CD8^+^ T cells, peripheral blood was collected from the orbital veins of the bm1 mice on days 5 and 10 (200 μL/mouse/time point). It was then processed into a lymphocyte suspension. Splenocytes were also collected on day 10 from each of the bm1 mice. Cells were then blocked with anti-mouse CD16/CD32 (clone 2.4G2) for 30 min and then incubated with a mixture of peptide-unloaded H-2K^b^-Ig dimers and PE-labeled anti-mouse IgG1 (clone A85-1) for 1 hour at 4°C. After washing, FITC-labeled anti-mouse CD8a and APC-labeled anti-mouse CD3e were added and the mixture incubated for an additional 30 min. Finally, cells were washed twice with 0.1 M PBS and analyzed by flow cytometry.

### Skin transplantation and treatment with killer MPs

Skin transplantation was performed as described by Garrod [38] with minor modifications. Briefly, dorsal tissue of ear were prepared from male C57BL/6 mice and then grafted onto the dorsal flank area of male bm1 mice under anesthesia. BAND AID^®^ styptic plaster (containing benzalkonium chloride) (Shanghai Johnson &Johnson, Ltd., Shanghai, China) was placed over the grafts, and an adhesive sterile bandage was applied for 7 days. Each grafted mouse was then housed individually. After removing the styptic plaster, all of the recipients for which the operation was successful were randomly assigned to one of four groups and injected (*via* the tail vein) with K^b^/killer-MPs, K^d^/killer-MPs, anti-Fas-MPs, or 0.1 M PBS on days 9, 11 and 13 post transplantation (1×10^7^ MPs/mouse/time point). The signs of rejection of grafts were monitored daily. Photographs of graft were taken every two days. Grafts were defined as rejected when less than 10% of the graft remained viable. K^d^/killer-MPs, a third-party alloantigen control, were prepared by co-coupling 10 μg of the anti-Fas mAb and 10 μg of peptide-unloaded H-2K^d^-Ig dimers onto 1 × 10^8^ PLGA MPs as described.

### Statistics

Statistical analyses were performed using GraphPad Prism 6.0 (GraphPad, La Jolla, CA, USA). All data are presented as the mean ± standard deviation (SD). Unpaired, two-tailed Student's t-tests were used to determine significant differences between groups. To determine the graft survival curve, a Kaplan-Meier graph was constructed, and a log-rank comparison of the groups was used to calculate the P values. *P* values < 0.05 were considered significant.

## SUPPLEMENTARY MATERIAL FIGURES


