# Supplementary material for: A biodegradable killer microparticle to selectively deplete antigen-specific T cells in vitro and in vivo
Source: Oncotarget. 2016 Feb 17;7(11):12176–90. doi: 10.18632/oncotarget.7519 (PMC4914277; doi:10.18632/oncotarget.7519)
Supplement: Supplementary file 1 [file oncotarget-07-12176-s001.pdf]

A biodegradable killer microparticle to selectively deplete antigen-specific T cells *in vitro* and *in vivo*

Supplementary Material

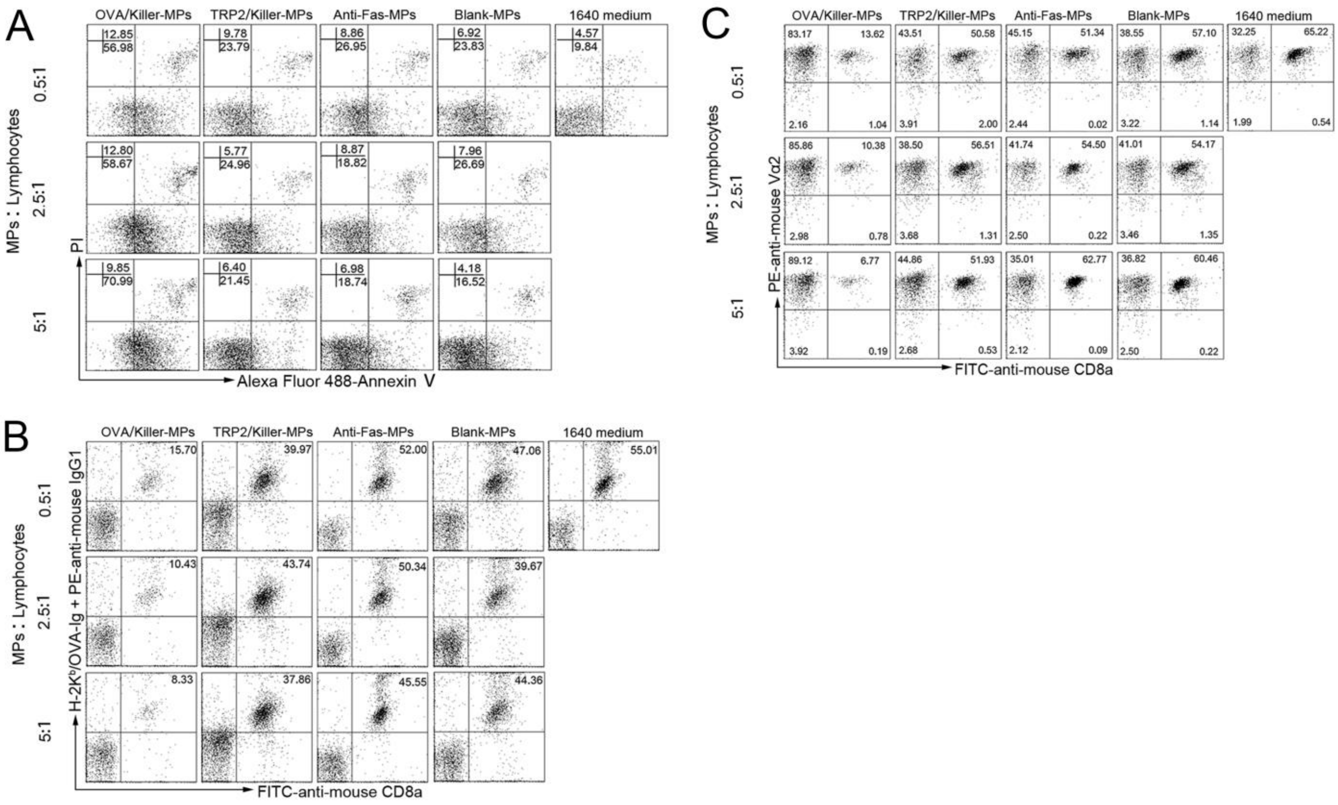

Supplementary Figure 1: *In vitro* elimination of OVA$_{257\text{–}264}$-specific CD8^{+} T cells by OVA/killer-MPs. (A) Representative flow cytometric dot plots for the apoptosis assay in Figure 3A. The dot plots are gated on the CD8^{+} T cell population. The proportion of apoptotic CD8^{+} T cells is shown in the top left quadrant. (B, C) Representative flow cytometric dot plots for the H-2K^{b} dimer staining in Figure 3B and for anti-mouse Vα2 TCR staining in Figure 3C. Dot plots are gated on the CD3^{+} T cell population. The percentage of double-positive cells is shown in the corresponding quadrant.

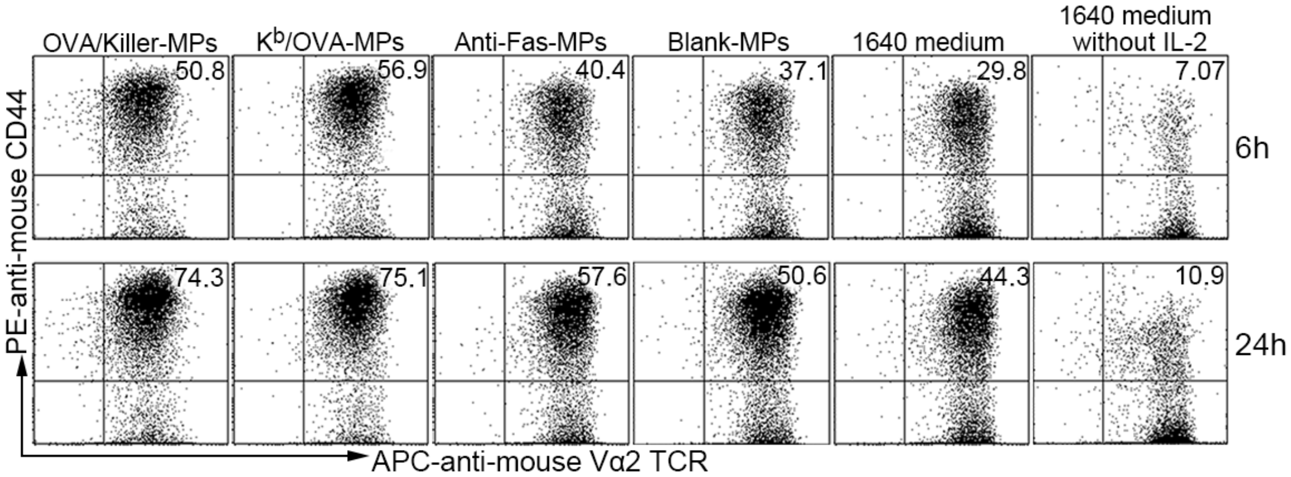

Supplementary Figure 2: Expression of CD44 on OVA-specific CD8^{+} T cells in co-cultures. Lymphocytes from OT-1 mice were co-cultured with OVA/killer-MPs, K^{b}/OVA-MPs, anti-Fas-MPs, or blank-MPs at a 1:1 ratio of MPs to lymphocytes in complete 1640 medium with IL-2. Lymphocytes incubated alone in complete 1640 medium without IL-2 were used as a control. After 6 and 24 hours, the cells were harvested and stained with PE-anti-mouse CD44, FITC-anti-mouse CD8a, and APC-anti-mouse Vα2 TCR and analyzed by flow cytometry. The data are representative of the results from three independent experiments. The dot plots are gated on the CD8^{+} T cell population. The percentage of CD44^{+}/OVA-specific CD8^{+} T cells is shown in the top right quadrant.

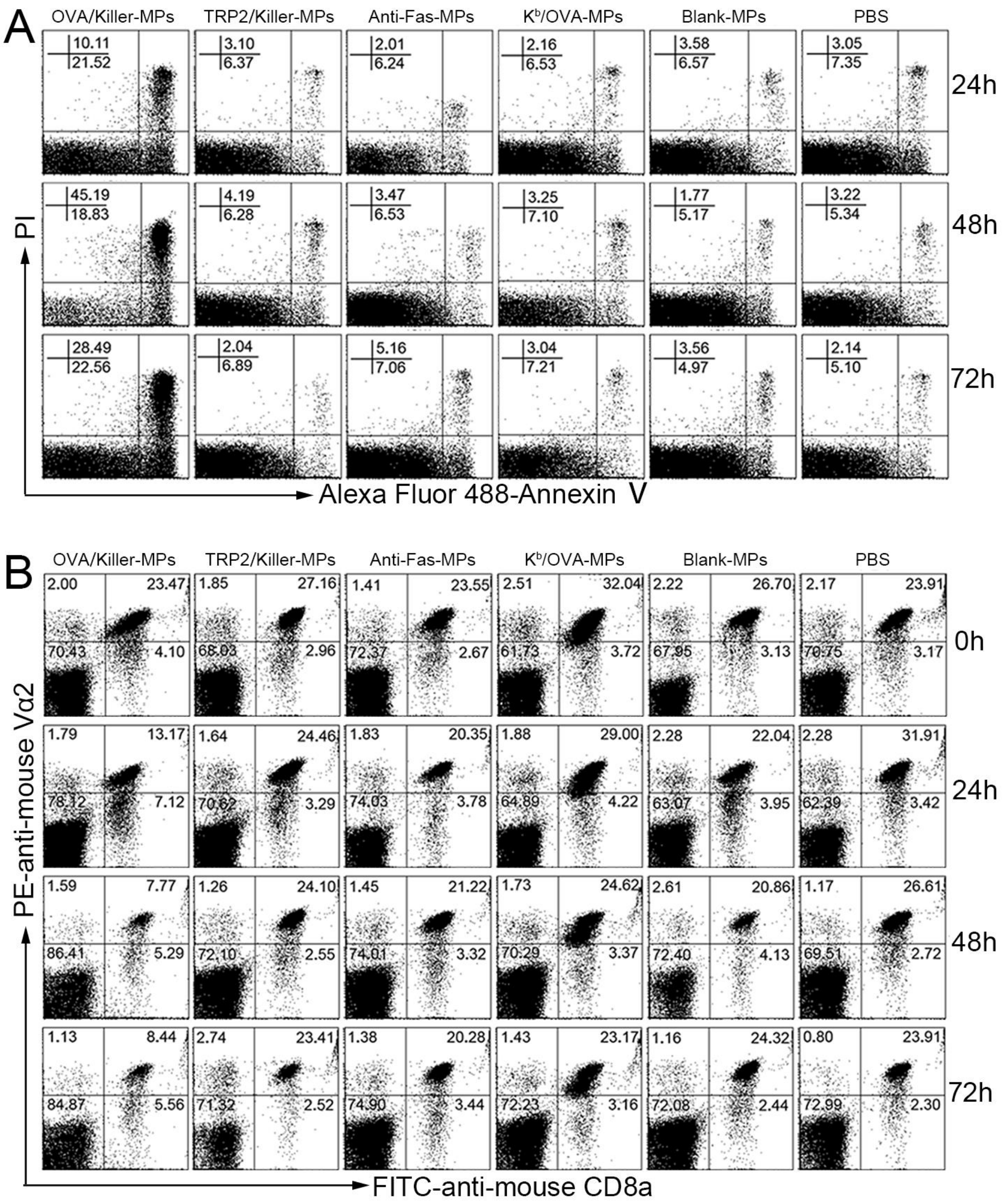

Supplementary Figure 3: *In vivo* depletion of $OVA_{257–264}$-specific T cells by OVA/killer-MPs. Representative flow cytometric dot plots for annexin V/PI staining of $CD8^+$ T cells in Figure 5B and anti-mouse Vα2 TCR staining of $CD3^+$ T cells in Figure 5C. The administration of OVA/killer-MPs elicited a strong apoptotic effect on $CD8^+$ T cells and a marked reduction of $OVA_{257–264}$-specific $CD8^+$ T cells among PBMCs. The percentages are shown in the corresponding quadrant.

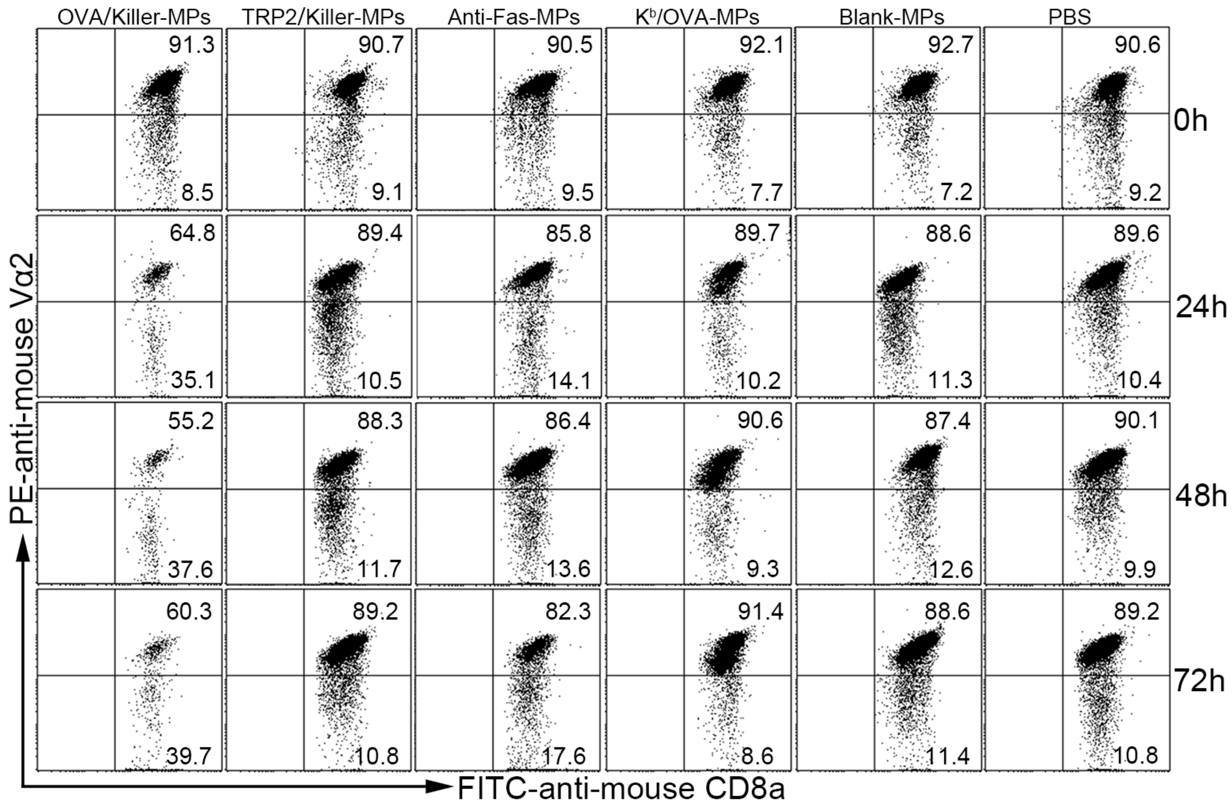

Supplementary Figure 4: *In vivo* depletion of $OVA_{257–264}$-specific T cells by OVA/killer-MPs. Representative flow cytometric dot plots for anti-mouse Vα2 TCR staining of $CD8^+$ T cells in Figure 5D. The administration of OVA/killer-MPs elicited a marked reduction of $OVA_{257–264}$-specific $CD8^+$ T cells among the $CD8^+$ T cell population. The percentages are shown in the corresponding quadrant.

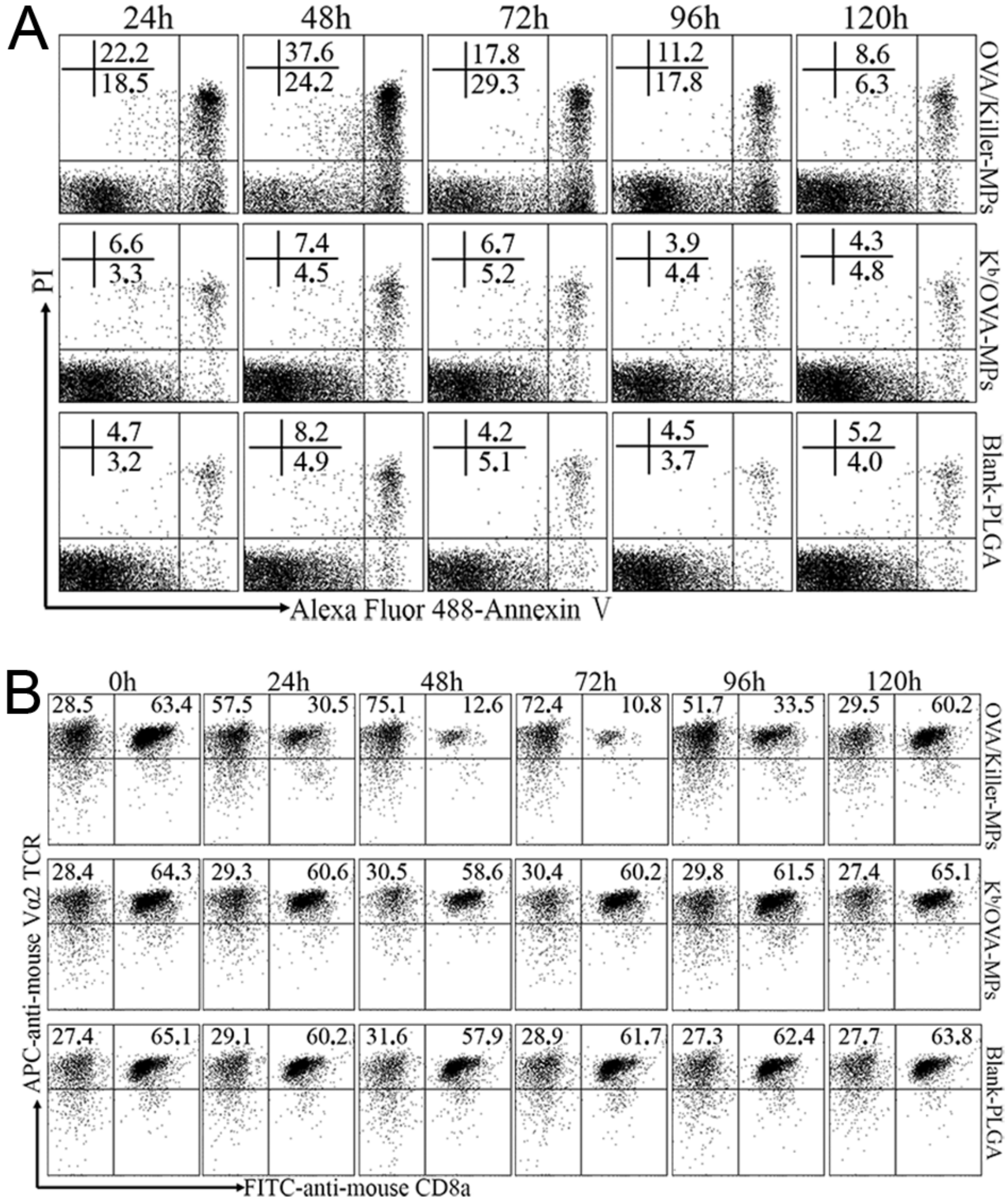

Supplementary Figure 5: Killing effects of killer MPs persisted for 4 days *in vivo*. OVA/killer-MPs, K^b/OVA-MPs, or blank-MPs were administered intravenously to OT-1 mice at 0 and 24 hours, and then monitored for 120 hours by detecting apoptotic $CD8^+$ T cells and the frequency of $OVA_{257–264}$-specific $CD8^+$ T cells at various time points. (A) Representative flow cytometric dot plots for the apoptosis assay in Figure 5E. The dot plots are gated on the $CD8^+$ T cell population. The percentage of apoptotic $CD8^+$ T cells is shown

in the top left quadrant. (B) Representative flow cytometric dot plots for the anti-mouse Vα2 TCR staining in Figure 5F. The dot plots are gated on the $CD3^+$ T cell population. The percentage of $V\alpha 2^+/CD8^+$ T cells is shown in the corresponding quadrant.
